# Intravitreal bevacizumab causes rapid regression of disc neovascularization associated with large optic disk melanocytoma: a case report

**DOI:** 10.11604/pamj.2024.48.156.44022

**Published:** 2024-08-06

**Authors:** Ogugua Ndubuisi Okonkwo, Adekunle Olubola Hassan, Arinze Anthony Onwuegbuna, Idris Oyekunle

**Affiliations:** 1 Department of Ophthalmology, Eye Foundation Hospital and Eye Foundation Retinal Institute, Lagos State, Nigeria,; 2 Department of Ophthalmology, Nnamdi Azikiwe University, Awka, Anambra State, Nigeria

**Keywords:** Melanocytoma, optic disc, neovascularization of the disc, retinal vascular occlusion, case report

## Abstract

Optic disk melanocytoma (ODM) is a rare ophthalmic tumor that can present with local compressive effects such as retinal vascular occlusion (RVO) that results in neovascularization of the disk (NVD) and is reportedly challenging to treat. We report the case of a 37-year-old Black African male with a two-year history of painless nonprogressive blur in his right eye vision. Findings on ocular examination include best corrected visual acuity right eye 6/18 and left eye 6/6, right eye relative afferent pupillary defect, and a large dark brown pigmented mass covering the optic disc measuring 4.8 mm x 4.6 mm. NVD was present along the superior disc margin and whitening of the superotemporal retinal vessels. Fluorescein angiography confirmed a superotemporal retinal vascular occlusion with non-perfusion and dye leakage from the NVD. Intravitreal bevacizumab and scatter retinal laser resulted in complete NVD regression within four weeks and no recurrence in 18 months. This case report suggests that earlier treatment with Intravitreal bevacizumab and scatter retinal laser photocoagulation is effective for the regression and stabilization of NVD associated with ODM. Aggressive ODM could be a feature in younger-age Africans. Yearly monitoring is required to detect an increase in ODM size that could suggest malignant transformation.

## Introduction

A melanocytoma of the optic disk is a pigmented tumor that originates from melanocytes of the optic disk and can extend into the peripapillary retina and the choroid [1]. In the past, melanocytoma was assumed to be a malignancy, but later evidence suggests its benign nature. In some cases, optic disk melanocytoma (ODM) is associated with features of local compressive changes, including ischemic optic neuropathy and central or branch retinal vascular occlusion [1-4]. Rarely, optic disk melanocytoma can transform into malignancy [5]. Furthermore, Thanos *et al*. reported a case of ODM and hemiretinal vascular occlusion with persistent optic disk neovascularization after adequate scatter retinal laser photocoagulation and two doses of intravitreal bevacizumab injection [4]. This case responded poorly to conventional treatment.

We report another case of optic disk neovascularization secondary to superotemporal branch retinal vascular occlusion from a large ODM in a 37-year-old Black African, that was responsive to one dose of intravitreal bevacizumab injection and scatter retinal laser photocoagulation to the superotemporal non-perfused area of the retina. Early intervention before the occurrence of vitreous hemorrhage was beneficial in our case.

This research followed the tenets of the Declaration of Helsinki. The patient presented in this case report gave a written, signed informed consent for publication of his case information and images. Ethical approval was obtained from the Eye Foundation health research ethics committee.

## Patient and observation

**Patient information:** a 37-year-old male was referred to our retina service for a two-year history of painless, nonprogressive blur in his right eye vision. He had no relevant past medical history and was in good health at presentation. He was not on oral or topical medication and did not ingest alcohol or tobacco. He had no contributory family history. He had no complaints in his left eye.

**Clinical findings:** his ocular examination revealed that the best corrected visual acuity (BCVA) of the right eye was 6/18 and could not be improved with refraction. BCVA in the left eye was 6/6, and other ophthalmic examinations were normal, except for the unremarkable finding of a dark pigmentary retinal degeneration in the retina periphery.

**Figure 1:**
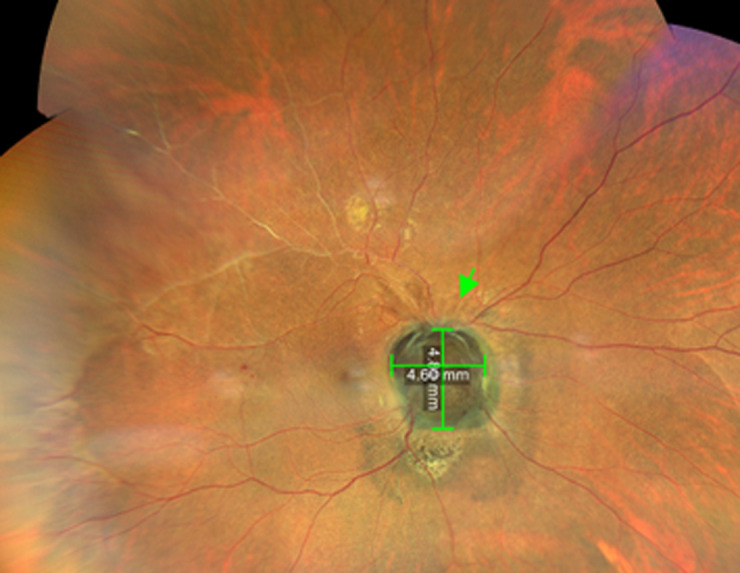
right eye fundus photography showing optic disk melanocytoma as a pigmented mass overlying the optic disk and the non-perfused superotemporal retinal vessels

**Figure 2:**
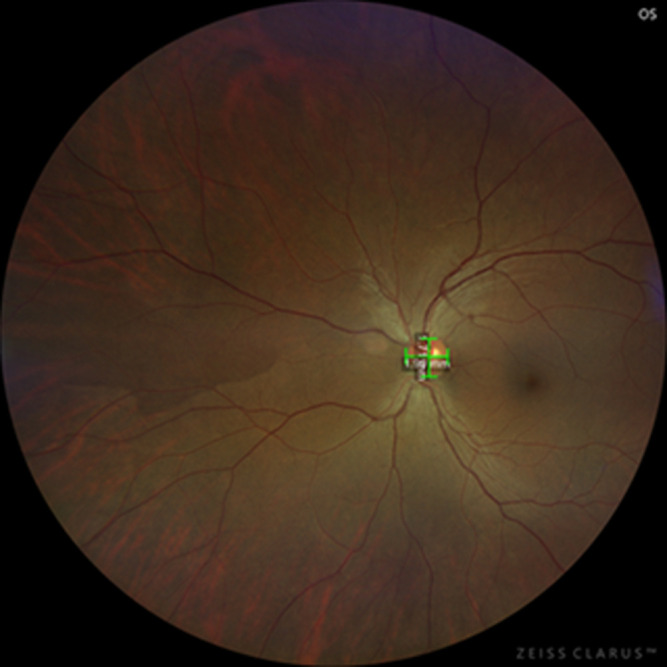
fundus photograph of the normal-appearing left fundus and optic disk

**Figure 3:**
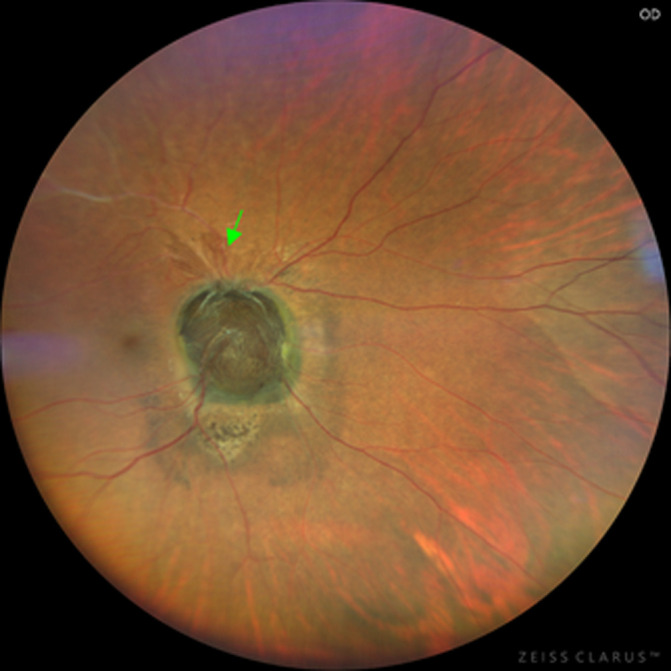
fundus photograph of the right eye showing neovascularization emanating from the superior aspect of the optic disk pigmented lesion

The findings in the right eye were an intraocular pressure of 12 mmHg and a relative afferent pupillary defect. There was no lens opacity, and the anterior segment was normal. On dilated fundoscopy, there was a large dark brownish pigmented mass lesion that covered the entire optic disk, extending into the vitreous, with the circumferential extension of pigmentation into the surrounding retina and involving the papillomacular bundle area, with pigmentary changes in the foveomacular, also extending superotemporal. The lesion measured 4.8 mm x 4.6 mm, vertical and horizontal diameter, respectively (Figure 1). The left optic disk diameter measured 1.7 mm X 1.9 mm (Figure 2). There was neovascularization on the superior aspect of the pigmented mass lesion, (Figure 3) (green arrow). Whitening of the superotemporal vessels and dot hemorrhage in the temporal macula was also observed (Figure 1). He was diagnosed to have right eye ischemic superotemporal vascular occlusion with neovascularization of the optic disk (NVD) secondary to large necrotic optic disk melanocytoma (ODM) based on the clinical findings.

**Figure 4:**
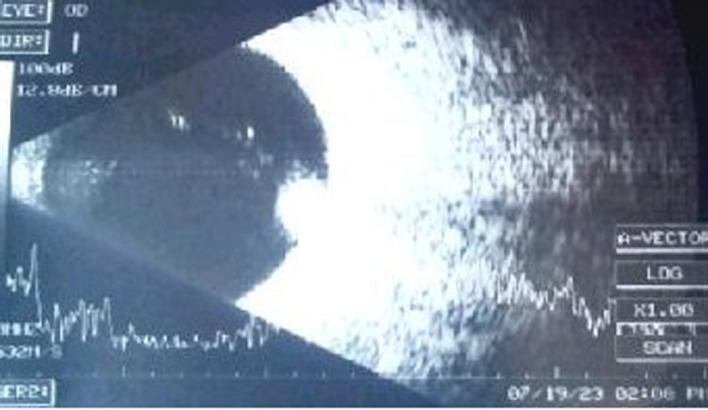
ultrasound B scan of the right eye shows a highly echogenic mass lesion protruding from the optic disc into the vitreous cavity

**Diagnostic approach:** the axial lengths of the right and left eyes were 23.15 mm and 23.44 mm, respectively. Right eye ultrasound B scan showed an elevated echogenic mass projecting into the vitreous cavity from the optic disk (Figure 4).

**Figure 5:**
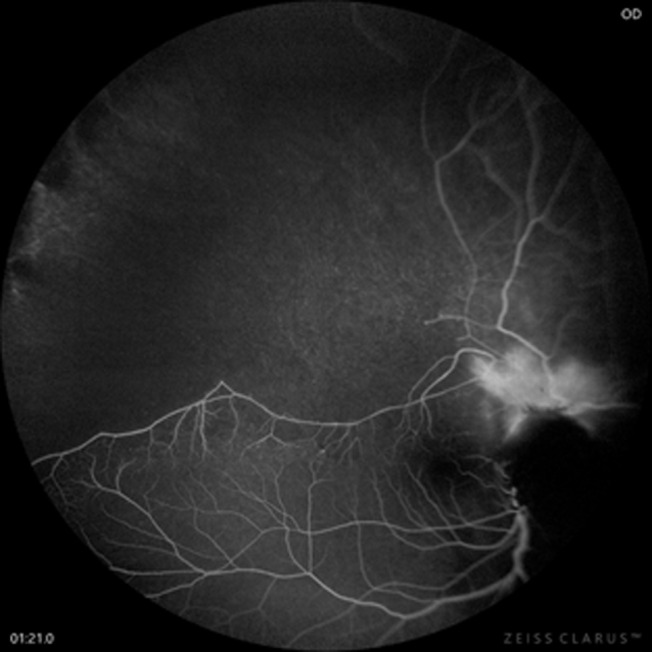
fundus fluorescein angiography venous phase showing peri optic disk hyperfluorescence due to dye leakage from the neovascularization on the disc and hypofluorescence in the superotemporal retina from non-perfused vasculature

**Figure 6:**
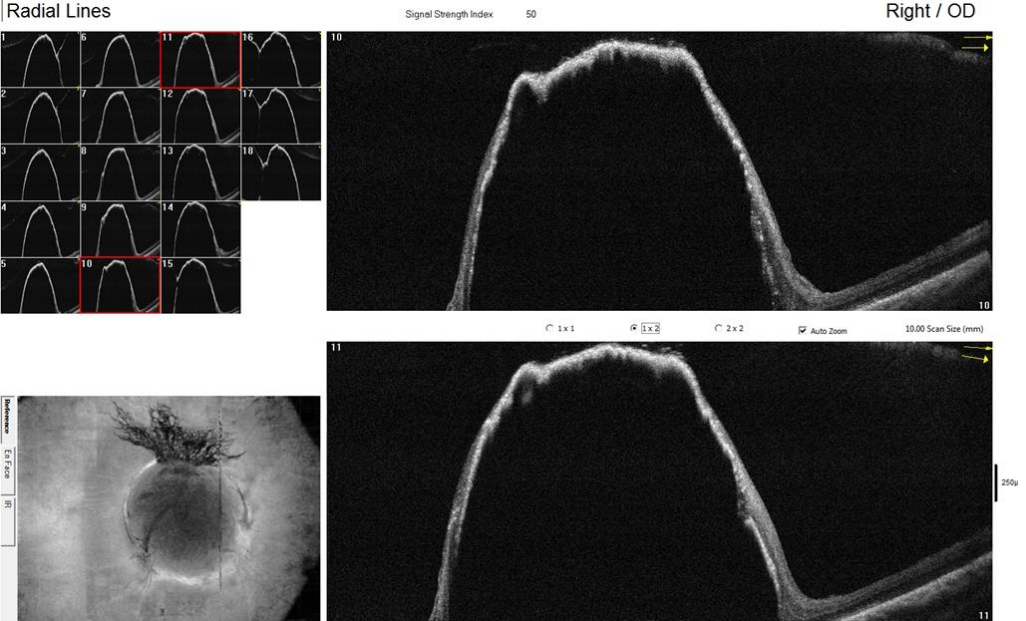
optical coherence tomography of the optic disk melanocytoma and adjacent retina shows the thinning and stretching of neural retina tissue at the base of the lesion and flattening at the top of the lesion

Fundus fluorescein angiography of the right eye revealed a wide area of non-perfusion (hypo fluorescence) in the superotemporal retina, with no dye transit in the superotemporal artery and vein (Figure 5). Neovascularization in the superior aspect of the optic disk appeared as increasing hyperfluorescence (dye leakage) on the late venous phase of dye transit. The perifoveal vascular complex surrounding the foveal avascular zone was irregular and ill-defined superiorly. The vasculature in the other areas of the retina was normal (Figure 5). Fundus autofluorescence (blue) of the ODM revealed hypo autofluorescence of the disc and a peripapillary ring of hypo autofluorescence that spared the fovea. Optical coherence tomography of the ODM and adjoining retina showed thinning and stretching of the adjacent retina along the base of the elevated mass lesion. The outline of the elevation was thinnest at the flattened top of the ODM (Figure 6).

**Figure 7:**
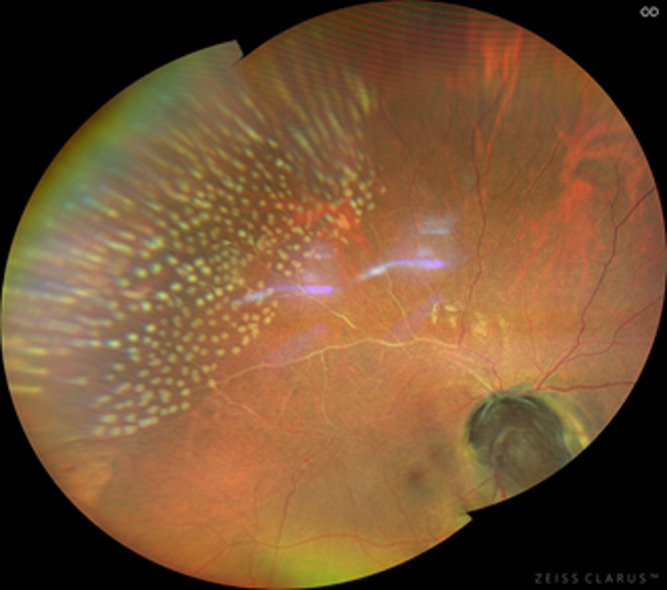
complete regression of the optic disk neovascularization, and retinal laser marks are evident in the superotemporal retina

**Therapeutic intervention and follow-up:** he received an intravitreal injection of 1.25 mg /0.05 cc bevacizumab two days after presentation and examination. When evaluated four weeks later, there was a complete regression of the NVD (Figure 7). There were no adverse effects or safety concerns following the intravitreal bevacizumab injection. His retina care was followed up with scatter retinal laser photocoagulation to the superotemporal non-perfused retina two days after his last clinical evaluation (Figure 7). His vision has remained stable with no recurrence of NVD for the past 18 months of follow-up care. Given the size and symptomatic presentation of the ODM, yearly examinations to assess for an increase in lesion size have been advised to examine for ODM growth and keeping in mind the possibility of malignant transformation.

**Patient perspectives:** the patient was well informed and involved in the decision-making of the treatment and was satisfied with the outcome of treatment. He has kept all scheduled clinic appointments and will be seen yearly to monitor for the growth of the ODM.

**Informed consent:** the patient gave written informed consent before each treatment and for the publication of his case report and images.

## Discussion

According to our search, there has been no report of NVD association with ODM in a Black African. Previous reports of neovascularization of the disk or choroid associated with ODM have been in Caucasians. Subretinal choroidal neovascularization has been reported by Urrets-Zavalia *et al*. Tran *et al*. and Kamisasanuk *et al*. [6-8]. In two cases intravitreal bevacizumab injection resulted in good outcomes and subretinal surgery resulted in improved vision in the case reported by Tran *et al*. [7].

Our case is younger than the three case reports mentioned previously and is a Black African. Shields *et al*. report another case of a Black African of similar age (35 years) as our patient who had similar presentation and signs of central retinal vascular occlusion [3]. In the case reported by Shields *et al*. the eye was eventually enucleated, and histopathologic findings revealed a large, necrotic melanocytoma of the optic disc and hemorrhagic necrosis of the retina secondary to obstruction of the central retinal artery and vein. It could be that ODM in younger Africans presents with a more aggressive disease, and this is open for further research.

Our report will be the second report of NVD associated with ODM. Thanos *et al*. reported a case of disc neovascularization associated with ODM following hemiretinal vascular occlusion [4]. In the reported case, the patient developed NVD after the vascular occlusion with deterioration of vision from hand motion (HM) to light perception (LP) and associated vitreous hemorrhage after conventional therapy using retinal laser photocoagulation and intravitreal bevacizumab. The treatment outcome of this case was poor. Our patient had NVD at presentation and did not develop a vitreous hemorrhage following treatment with intravitreal bevacizumab. Other cases of vascular occlusion associated with ODM have been reported, including central vascular occlusions. Interestingly, NVD developed in Thanos *et al*. [4] patient with hemiretinal and our patient with a branch vascular occlusion, but not with more widespread occlusions such as central retinal vascular occlusions [3]. However, rubeosis has been reported with central vascular occlusion [1]. Retinal vascular occlusion due to ODM results from ischemic necrotic changes within the ODM or vascular compression.

The NVD that appears in ODM after vascular occlusion is mediated by vascular endothelial growth factor (VEGF) induced mechanisms since our case responded promptly (with regression of NVD) to a single injection of intravitreal anti-VEGF, bevacizumab. Our case is unlike Thanos *et al*. which responded poorly to both intravitreal bevacizumab injections and retinal laser photocoagulation and eventually suffered a complete loss of vision. Though vision in our case was reduced at presentation, there was no further deterioration during the evaluation and treatment period and vitreous hemorrhage was not a feature. He had been symptomatic for two years and preserved reasonable vision. In our case, we theorize that decreased vision is related to macular ischemia, which is evident using fluorescein angiography. Macula and optic disc optical coherence tomography angiography (OCT-A) images were of poor image quality, with presence of significant artifacts. However, swept-source OCTA, which has more extended depth penetration, has been used to evaluate ODM, offers novel diagnostic utility, and helps differentiate ODM from melanoma [9]. Fundus autofluorescence (FAF) showed an absence of autofluorescence on the disc, normal foveomacular fluorescence, and a ring of hypo autofluorescence within the limits of the pigmentary lesion in the peripapillary retina. Another possible reason for loss of vision in our case could be compression of the nerve fibers within the optic disc. Since the ODM lesion size was large, intra- and peri-optic nerve extension and compressive mass effect would damage the optic disc nerve fibers, including the fibers of the papillomacular bundle. Optic disk compression also explains the right eye relative afferent pupillary defect present on clinical examination.

In the context of differential diagnosis of NVD in ODM, there is a report of angle neovascularization that developed from severe retinal ischemia following vascular occlusion that evolved into rubeotic glaucoma [10]. Other NVD-related retinovascular causes, including retinal vein occlusion and proliferative diabetic retinopathy, should be considered. Pigmented optic disk lesions as differentials include choroidal melanoma, choroidal nevus, metastatic melanoma of the optic disc, and combined hamartoma of the retina and retinal pigment epithelium (RPE).

It was possible to objectively delineate the size of the mass using ultrasound B scan and fundus photography. These baseline measurements will be helpful during further evaluations and determination of lesion growth in future examinations. Lastly, though the diagnosis of ODM is primarily clinical or clinicopathological, multimodal imaging helped determine the lesion's extent, effect, and response to treatment.

## Conclusion

This case illustrates the outcome of intravitreal bevacizumab for the treatment of a rare complication (optic disk neovascularization) of a rare tumor (optic disk melanocytoma). A previous report on the use of intravitreal bevacizumab to treat a similar case presentation resulted in poor outcomes. We show that a more favorable outcome can be achieved if treated at an earlier stage of the disease. Though neovascularization of the optic disk associated with ODM is rare it can occur secondary to vascular occlusive disease in a relatively younger African. Vascular endothelial growth factor-mediated mechanism, as in other retinal vascular diseases, is responsible for ODM-associated NVD and responds to intravitreal anti-VEGF. Intravitreal anti-VEGF treatment should preferably be administered before the occurrence of secondary vitreous hemorrhage and other troubling consequences of NVD including rubeosis. The younger age of black Africans with more aggressive ODM disease requires further research.
